# Sustainable Dyeing and Mordanting of Silk with a Binary Colorant from Agro-Waste Material

**DOI:** 10.3390/molecules31183253

**Published:** 2026-09-14

**Authors:** Shahid Adeel, Muhammad Kamran, Muhammad Afzaal, Muhammad Aftab, Khurram Shahzad Munawar, Asfandyar Khan, Fiaz Hussain

**Affiliations:** 1 Department of Applied Chemistry, Government College University Faisalabad, Faisalabad 38000, Pakistan; 2 Department of Food Science, Government College University Faisalabad, Faisalabad 38000, Pakistan; 3 Department of Statistics, Government College University Faisalabad, Faisalabad 38000, Pakistan; 4 Department of Chemistry, University of Mianwali, Mianwali 42200, Punjab, Pakistan; 5 Department of Textile and Apparel Science, School of Textile and Design, University of Management and Technology, Lahore 54770, Pakistan; asfandyar.khan@umt.edu.pk; 6 Department of Textile Engineering, Daffodil International University, Dhaka 1216, Bangladesh; 7 Department of Mechanical Engineering, Gachon University, Seongnam-si 13120, Gyeonggi-do, Republic of Korea

**Keywords:** C.C.D., cotton, harmal, myrobalan, natural dyeing process, response surface methodology, sustainability, thuja

## Abstract

The use of agro-waste materials as an alternative to synthetic dyes has gained considerable interest in the development of sustainable textile coloration. This study investigates the utilization of binary colorants from agro-waste sources such as harmal seeds and thuja leaves for silk, with statistical selection of variables. A central composite design (C.C.D.) coupled with microwave radiation was employed to select the significant dyeing conditions for excellent yield. It has been found that the pH 3 extract obtained from 6 g of binary powder after irradiation for 3 min, when applied at 75 °C for 40 min, produced an excellent yield of up to 6.15 (K/S). Two-way ANOVA also revealed the significance of selected dyeing variables for silk coloration using a binary colorant. The use of optimum amounts of chemical and bio-mordants produced colorfast, stable shades with fastness ratings of 4/5-5 for light, washing, and rubbing. The improvement in functional properties such as antioxidant and antibacterial properties of both the extracts and dyed fabric after MW treatment reveals that this bi-colorant has potential benefits for the community. It has been concluded that sustainable mordanting of silk has added more value in the coloration of silk with binary colorants by producing colorfast shades upon dyeing using statistically selected variables.

## 1. Introduction

A range of industries, especially textiles and its allied sectors, are under increasing pressure to adopt sustainable and green approaches due to strict regulations and increasing demand for zero-emission effluents. Conventional textile dyeing and printing, involving the use of toxic synthetic dyes and chemical auxiliaries, can cause serious threats to humans, the environment, and aquatic life [1]. The manufacturing and industrial applications of synthetic dyes often involve the use of auxiliaries, and they produce carcinogenic by-products that cause serious concerns and cause serious health issues such as asthma, cancer, skin allergy, etc. [2]. So, environmentally friendly and sustainable natural dyes as an alternative to carcinogenic synthetic dyes have gained considerable attention from researchers in academia and industry working in the fields of textile science and allied fields [3]. Plant-based natural dyes have found a strong position as promising alternative dyeing materials due to their unique features, including sustainability, renewability, and their environmentally friendly nature. These natural dyes are generally extracted from agro-waste, and they provide additional features such as antiviral, antioxidant, anti-inflammatory, antibacterial, and antifungal properties [4]. These colorants are ambassadors of cultural heritage, so they can be used in many regular household items such as handicrafts, artwork, artisan work, etc. Thus, the revival of biological coloring, along with the many benefits of using natural dyes in textiles and the allied food, flavor, cosmetic, pharmaceutical, staining, and leather industries, is on the way [5].

However, besides the versatile nature and advantageous characteristics of these dyes, poor extraction yield, low colorfastness, and dye fixation issues limit their commercial applications. To address these challenges, many efforts have been made to explore innovative, cost-effective, short-duration, and energy-efficient sustainable technologies [6]. Among the various strategies, microwave (MW) treatment has gained considerable attention due to its energy efficiency and cost-effectiveness. These rays help to improve the surface of textile substrates to retain dye molecules to the maximum extent, resulting in enhanced color strength. These rays also work in energy-efficient isolation and dyeing systems by saving cost through effective solute–solvent interaction for effective results [7]. Simultaneously, statistical modeling tools like response surface methodology (RSM) and central composite design (CCD) have emerged as innovative and effective technologies for improving process optimization and product development with excellent results [8]. These innovative approaches play a pivotal role in process efficiency by determining the possible effective interactions between parameters to maintain product quality, resulting in fewer experiments and a more cost-effective, energy-efficient dyeing process [9,10]. Hence, the combined effects of statistical modeling for process optimization and MW treatment for product development have the potential to give promising results [11,12].

In addition to efficient dye extraction and process optimization, the use of bio-mordants is increasing to improve the colorfastness properties of the dyed substrates [13]. These mordants (inorganic or organic) are usually used before and after dyeing of fabric to improve shade quality and quantity [14]. Currently, tannin-based bio-mordants are being used as an alternative to toxic metallic mordants in the natural dyeing process to make the dyeing process greener, more sustainable, and environmentally friendly [15]. Despite various studies reporting on the utilization of single-plant extract-based natural dyes for textile dyeing, there are limited studies available that utilize binary plant extract-based natural dyes for textiles, especially silk dyeing. Furthermore, the MW-assisted extraction of plant dyes and processing parameter optimization through RSM for sustainable and efficient natural-dye textile coloration systems, while utilizing green mordants, are largely unexplored [16]. So, there is a dire need to explore binary natural dye systems for sustainable, energy-efficient, and low-cost textile dyeing processes to address this significant research gap. To the best of our knowledge, there is not any comprehensive study that reports MW-assisted, RSM-based optimized processing parameters for the binary-system-based dyeing of silk using thuja and harmal extracts [17].

So, the present research work reports an innovative and sustainable silk dyeing technique using new sources of dyes as a binary mixture. The first source is thuja, commonly known as the tree of life, and the other is harmal, commonly known as esfund or wild rue. Thuja (*Thuja occidentalis*), being a member of the cypress family, is an evergreen tree that has many phytochemicals, where flavonoids have been found to act as bio-dye for fabrics and yarns. Its extract is used to cure respiratory, viral, and bacterial diseases, immunity, asthma, influenza, etc. [18]. Harmal seeds (*Paganum harmala*) have many phytochemicals, where alkaloids are responsible for their coloring. Its extract helps to improve neuro and hepatic issues, arthritis, inflammation, etc. [19]. By combining their extracts, the joint effect of alkaloids from thuja and flavonoids from harmal with optimized dyeing conditions has the potential to provide a soothing shade and good colorfastness on the silk fabrics [20]. Silk was selected as a textile substrate due to its good tensile strength, lustrous appearance, less flammability, excellent dye affinity, and presence of abundant amino and amid functional groups, which are capable of having strong interaction with the natural dyes. The functional hydroxyl groups of natural dyes develop a strong interaction with the amino and amid functional groups of the silks, resulting in good colorfastness [21,22]. The novelty of this research is the integrated use of harmal seeds and thuja leaves as a binary agro-waste-derived colorant for silk, along with MW-assisted extraction, statistical optimization of multiple processing variables, including time, temperature, pH, etc., and sustainable mordanting. Compared to conventional research works focusing on a single plant colorant or individual processing variables, the present work combines color-strength optimization with fastness and functional-property evaluation, resulting in a more comprehensive, sustainable, and reliable route toward multifunctional and resource-efficient natural silk coloration.

To the best of our knowledge, limited studies have been done on the isolation and usage of binary colorant materials separately and in combination; however, the use of combined extracts from dried thuja leaves and dried seeds of harmal for an eco-friendly silk dyeing framework using a statistical approach and MW radiation with green mordanting has not been done so far. The key objectives of this study are to utilize agro-materials as binary natural dyes, enhance dye extraction yield, optimize coloration parameters, improve dye uptake, and improve the colorfastness of silk fabrics with sustainable green mordants, while minimizing the environmental impact [23]. For the first time, in this innovative integrated binary dyeing system, a response surface method (RSM) developed through central composite design (CCD) in combination with MW treatment has been used to select dyeing variables to make the coloration process cost-effective and time- and energy-efficient by reducing the number of experiments. The unique findings of our work could be a great step towards sustainable textile processing and the development of highly efficient natural dyeing technologies that are suitable for versatile industrial applications [24].

## 2. Material and Methods

### 2.1. Collection of Material

Dried harmal seeds and dried thuja leaves were pulverized finely and screened up to 20 mesh to be used in the isolation process. A tannin-based bio-mordant, such as dried powder of myrobalan, was also pulverized, screened, and stored for further coating to develop colorfast shades. The commercially available non-toxic electrolytes such as Al^3+^ salt (Al-acetate) and Fe^2+^-salt (green vitriol) of 99% purity were purchased from Sigma-Aldrich (St. Louis, MO, USA). Lab-grade sodium hydroxide (NaOH) and hydrochloric acid (HCl) of 99% purity were also purchased from Sigma-Aldrich. All the chemicals were stored and used without any further purification. The 60 GSM (grams per square meter) silk fabric was purchased from the local textile market, washed with soap for 15 min with normal warm water, dried, and stored to be used further for the dyeing process.

### 2.2. Statistical Selection of Variables

For the selection of radiation time, powder amount, contact variables (time and temperature), and dye bath pH, the statistical approach (C.C.D.) under the surface response method (RSM) was taken, where after formulation of the design, each experiment was carried out, and the corresponding results were determined and analyzed [25]. By following the design, 2, 4, 6, 8, and 10 g of binary powder (1:1 = thuja:harmal) was mixed with 100 mL of water, boiled for 1 h, and filtered. The microwave (MW) treatment of the binary mixture and silk fabric was given for 1–5 min using a high-power microwave radiator as per the design instructions. The pH of the extracted binary colorant was adjusted upto 3 before application on the silk. To adjust the pH of binary extracts, the required volume of an already prepared alkaline/acidic solution (0.1 M NaOH/HCl) was added by occasionally stirring it into the mixture. A series of experiments, as given in Table S1, were performed, and the results evaluated in terms of color strength (K/S) were assessed using two-way ANOVA. The detailed scheme has been presented in the schematic flow sheet (Figure S1).

### 2.3. Non-Toxic Coating for Shade Development

New shades were obtained using binary colorants from harmal and thuja powder extract under selected conditions by coating the silk fabric before (pre) and after dyeing (post) under statistically selected conditions. Keeping the non-toxic additive (chemical mordant)-to-fabric ratio 25:1, before dyeing each sample was treated with 0.5–2.5 g/100 mL of Al-salt and Fe-salt solution at 60 °C for 45 min and then dyed under statistically selected conditions [26]. The same process of chemical coating was done after dyeing of the silk with the binary colorant under selected conditions called post-mordanting. Furthermore, plant extracts such as myrobalan and pomegranate, acting as green anchors with bioactive compounds (tannin) were introduced before and after dyeing of the silk at statistically selected conditions. For this purpose, extracts were prepared by mixing 0.5 to 2.5 g of each pulverized powder with 100 mL of water and filtered, and 25 mL of each filtrate was applied, keeping a bio-mordant-to-fabric ratio of 25:1.

### 2.4. Analysis of Fabric and Extract

The antioxidant activity using the DPPH assay and antibacterial activities using *E. coli* and *S. aureus* strains for the extract and fabric have been assessed using standard protocols by following documented methods [27,28,29]. To assess the color strength (K/S) through the computed Kubelka–Munk Equation and the tonal variations in the dyed fibers with the computed CIE Lab and CIE LCh system color spectrophotometer (CS-410, Shanghai, China) computed with a D65 10° observer was used. Fourier Transform Infrared (FTIR) spectroscopy was used to evaluate the presence of functional groups in the binary colorants, silk fabric, and dyed silk fabric. By following ISO standards, the colorfastness to washing as per standard protocols of ISO105 CO3, colorfastness to light exposure as per the given standard methods of ISO105 BO2, and colorfastness to rubbing were evaluated as per the standard procedures of crocking of ISO105 X-12 [26]. For clarity and ease of understanding, a technical step-by-step experimental flowchart is shown in Figure S1.

## 3. Results and Discussion

Day by day, the role of statistics is rising in process and product development for the selection of variables. These methods help in the industrial sector not only to save energy, time, and cost, but also by their use in modeling large-scale series of trials using only a single run [30]. Among such methods, central composite design through surface response methods has gained importance in the industrial sector. The use of this design for a bio mixture of thuja and harmal has been observed. Analysis after performing a series of experiments reveals that the model is fit and linear at a 1% level (*p* = 0.01). Using a central composite design, the inclusion of microwave radiation for stimulation of the binary extract made the dyeing process more eco-friendly and cost-effective. Results show that exposure of the extract from 6 g of powder on fabric for 1 min gives a dyeing strength up to 3.49 (K/S), and exposure up to 5 min followed by dyeing of silk provides an even better strength (K/S = 4.68). However, if the extract from 6 g is irradiated for up to 3 min, then dyeing of the silk with the binary extract provides a shade of excellent strength (K/S = 6.15). This provided excellent observed results, showing significant yields, and MW treatment was both significant and relevant for developing a shade of the highest strength (Table 1). Statistically (Table S2), the role of radiation has been found significant at the 1% level (*p* = 0.01), whereas its role in combination with powder amount is significant at a 2% level (*p* = 0.02). This interaction has worked well, as MW rays transfer energy into solvent molecules, and these high-energy solvent molecules interact with plant materials and effectively extract the color from them [31]. The biological materials exist in the form of clusters, and these rays break into small molecules. These small colorant molecules are entered into voids of the surface-modified silk fabric up to the maximum level of extract (K/S = 6.15). A low powder amount can show higher yield, whereas a powder amount that is too high allows other biomolecules to be isolated alongside the colorant, which also causes aggregation on silk fabrics. On dyeing, either colorant in the form of aggregates remains at the surface, or other by-products show dominant behavior, causing low yield (K/S = 4.18) [32]. Here, for isolation, 6 g of binary powder is optimal, and for radiation, exposure up to 3 min is the best condition to get a high yield onto silk. The nature of the extract (pH), depending upon the fabric used and its original form, also adds value in dyeing. As silk is a protein in nature and has dual binding properties, under acidic conditions silk fabric develops a strong interaction with the dye molecules [33]. However, a condition that is too acidic or too alkaline can damage the silk fibers, which cannot hold dye molecules firmly to provide a high yield. After surface upgradation by MW treatment up to 3 min, the use of binary extract at pH 3 provided a shade of high strength. At pH 7, the dyeing of the silk after a 3 min exposure with a binary extract of 6 g of thuja and harmal gave the lowest yield (K/S = 1.08). Statistically, it can be seen that the role of pH (pH 3) in silk dyeing has been found to be highly significant. Similarly, the role of radiation with the binary extract, pH, and powder amount is also highly significant (*p* = 0.006). The contact time of the silk with the binary colorant at a particular heat level for a particular duration always affects dyeing quality. Here, MW treatment of both the extract and fabric improves dyeing quality by reducing these contact points. The results show that after MW treatment for up to 3 min on both the fabric and the binary colorant of 3 pH, made from thuja and harmal powder (6 g), the dyeing of silk at 75 °C for 3 min gives the highest yield (K/S = 6.15). The dyeing of the treated fabric with the binary extract at a low temperature (55 °C) provides a yield of up to 4.54 (K/S value), while at 95 °C, the dyeing of silk with the binary colorant provides a very low yield (K/S = 3.64). Silk is a very compact and versatile fabric wherein the fibers are only partly active and require a high temperature for binding with colorant molecules. During dyeing at lower temperatures, the binary colorant molecules are not accelerated enough to bind firmly. Similarly, very high temperatures cause either damage of fibroin fibers or degradation of dye. In both cases, due to poor interaction of fewer molecules with weak fibers, a low yield is found. A similar situation can be seen with the duration of dyeing [1,34]. A short dyeing time can cause less sorption of dye molecules on the fabric or the movement of a smaller amount of binary colorants towards the fabrics. In both cases, the aggregates remained unaffected during washing followed by drying, and analysis shows lower yields (K/S = 4.33). If the dyeing duration is too long, e.g., if contact heat is supplied (75 °C) for 60 min, the binary colorants may face degradation or may favor the rate of desorption, thereby leading to a low yield (K/S = 5.10). However, for the pH 3 binary extract after MW treatment for up to 3 min, when used to dye silk at 75 °C for 45 min, the highest yield is found (K/S = 6.15). In the statistical analysis, it can be seen that the interaction of radiation with time (*p* = 0.08) and with temperature (*p* = 0.001) is significant. Similarly, selected time and temperature (*p* = 0.01) and pH (*p* = 0.009) conditions are highly significant. pH and radiation time are found to be significant color parameters among the proposed conditions (Table S2). All interaction effects are found to be significant except powder*temperature and radiation time*time. The adequacy of the fitted model is observed as approximately 90%, along with a low standard deviation (S = 0.546469), which ensures the reliability of the experimental work.

**Figure 1:**
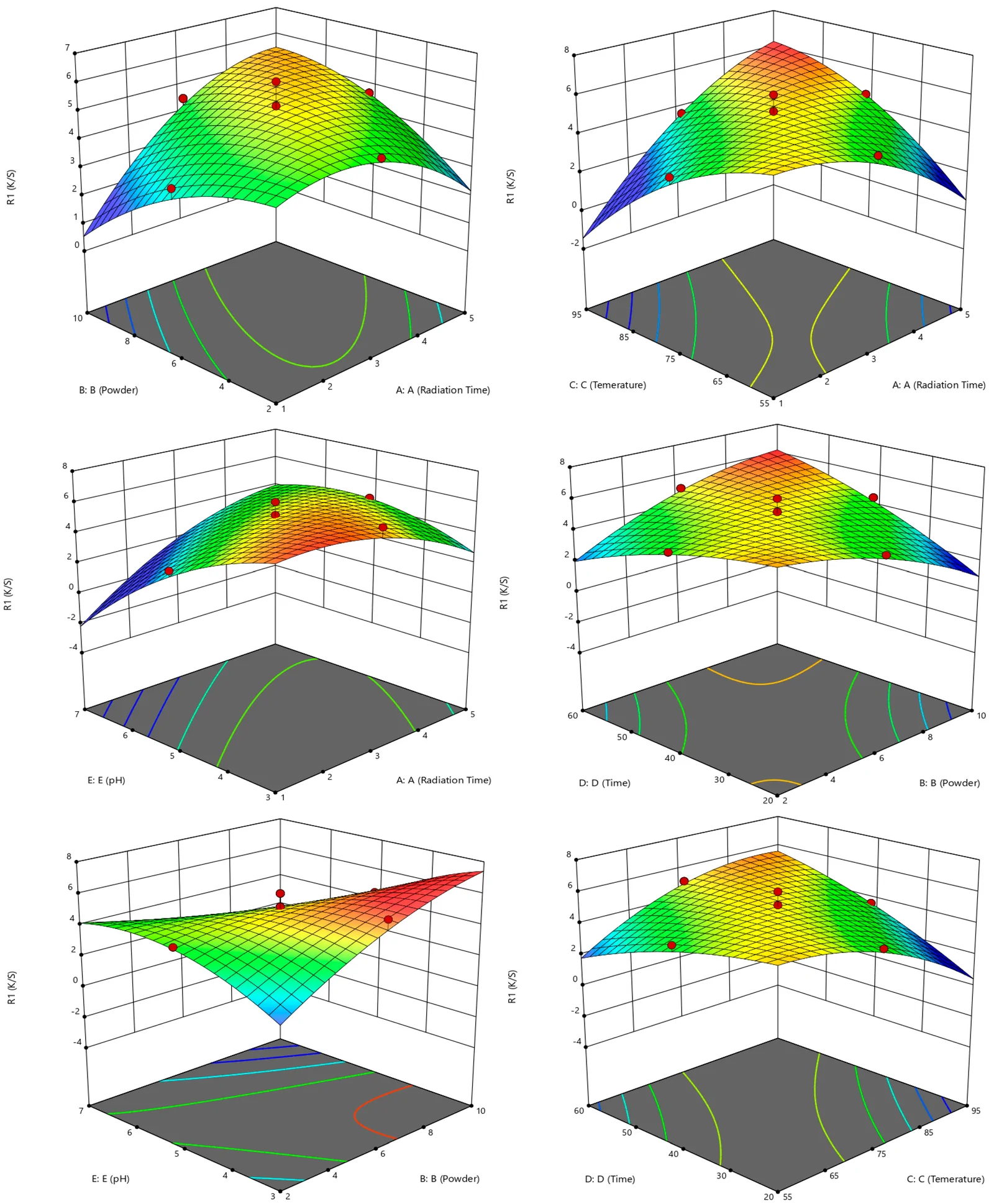

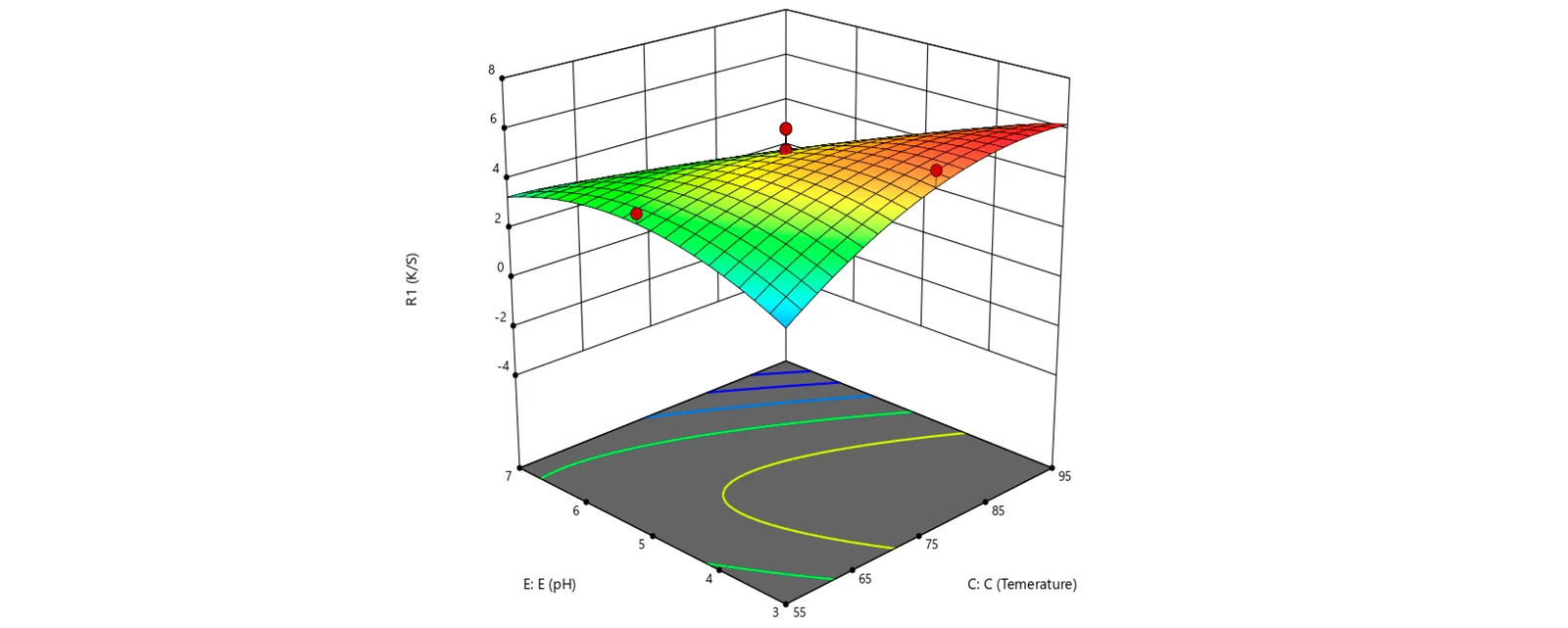
Graphical display of interaction effects using 3D surface plots.

**Figure 2:**
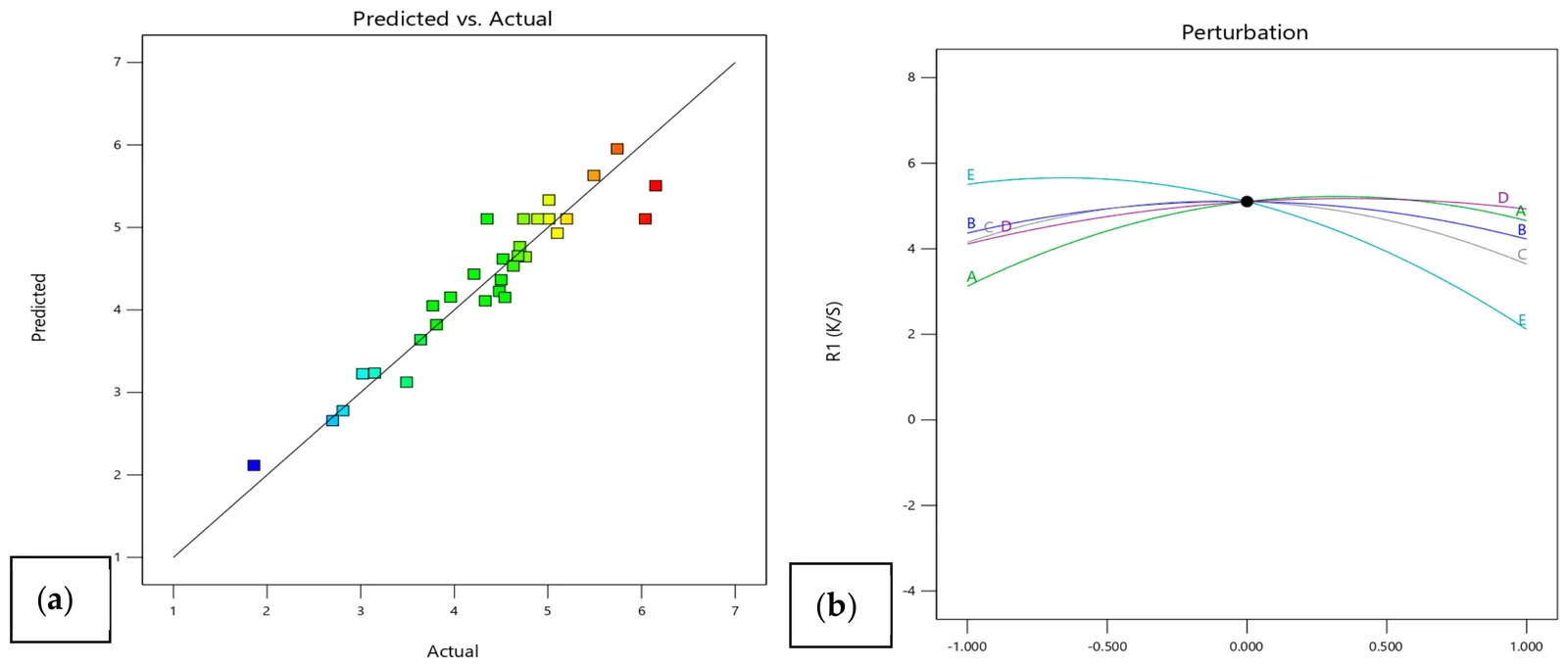
Model adequacy plot (**a**) and most-influential-factor (perturbation) plot (**b**).

This result is also illustrated in the 3D surface plots (Figure 1). The plots indicate good agreement between the predicted and experimental results (Figure 2a,b). This suggests the model has good accuracy and predictive capability. The apparent role of pH shows the steepest curve and largest deviation from the reference point, indicating it is the most influential factor for getting the highest yield. Factors such as radiation time and dyeing temperature show moderate influence, while dyeing time and powder amount have relatively smaller effects on the response. Hence, both time (40 min) and temperature (75 °C) played a significant role in the dyeing of silks with binary colorants from thuja and harmal after the MW treatment of up to 3 min.

**Figure 3:**
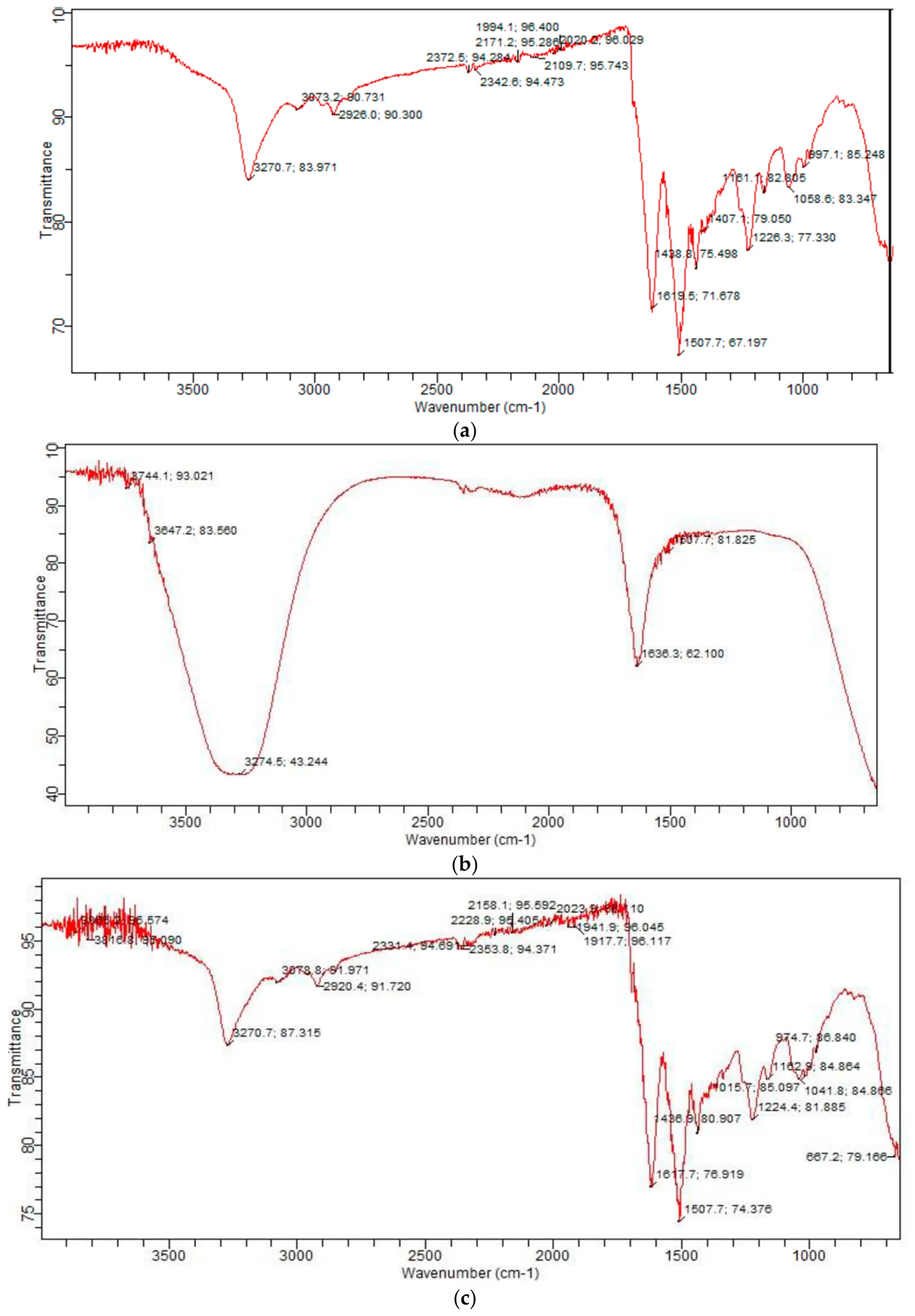
(**a**) ATR-FTIR interpretation of silk fabric. (**b**) ATR-FTIR interpretation of binary extract from harmal and thuja. (**c**) ATR-FTIR interpretation of dyed silk fabric using harmal and thuja.

**Figure 4:**
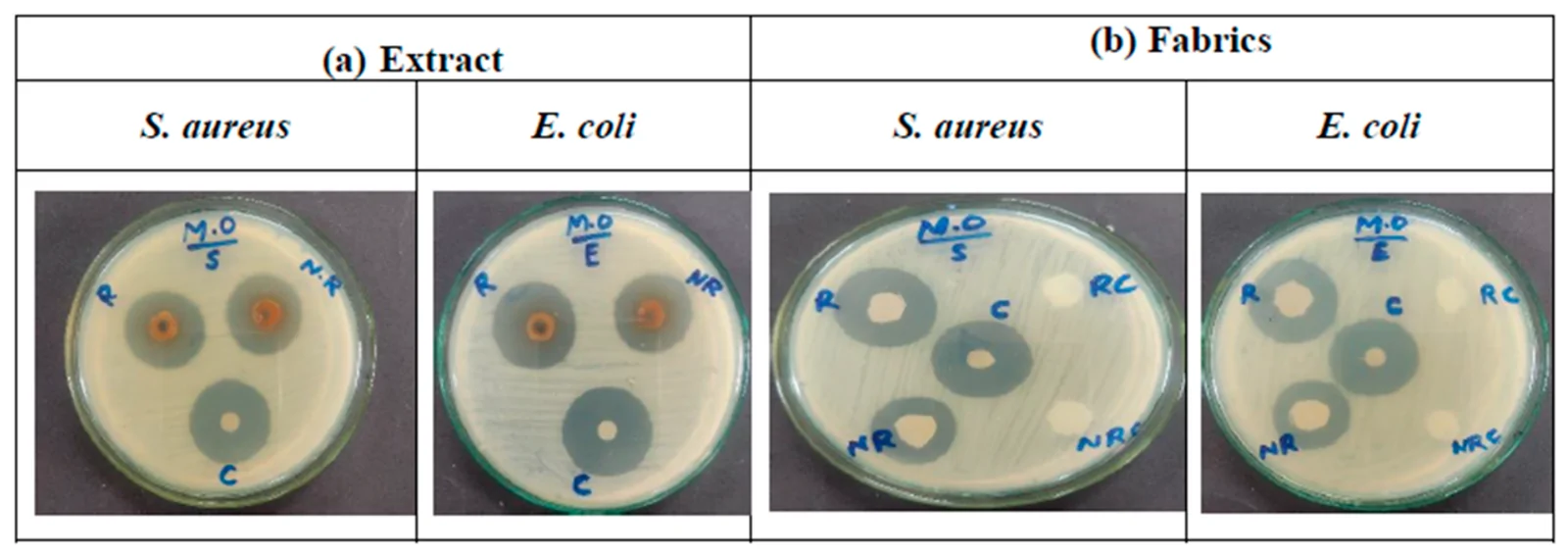
Antibacterial activity of non-irradiated and irradiated binary extract of thuja and harmal (**a**) and of non-irradiated and irradiated dyed fabric with binary extract of thuja and harmal (**b**).

The FTIR spectra presented in Figure 3a–c for the silk fabric, binary extract, and dyed silk reveal the existence of functional groups and their interaction. The functional peaks for silk represent amido groups, i.e., amide I–III (1250–1650 cm^−1^), C=O (near about 1650–1600 cm^−1^, and -NH (3200–3300 cm^−1^). Similarly, the binary extract spectral images represent the presence of CH functional groups (2800–2900 cm^−1^), OH groups (3200–3600 cm^−1^), and -NH groups (3100–3500 cm^−1^) by harmal alkaloids, an aromatic conjugation system by flavonoids from thuja leaves (1650–1500 cm^−1^), and the -C=O group of flavonoids (1600–150 cm^−1^). Thus, the functional groups showing characteristic intensities reveal the presence of functional sites in the silk, i.e., amido linkage, alkaloids by harmal, and flavonoids by the thuja leaves [35,36,37,38,39,40]. The results given in Figure 4 for the antibacterial action of the fabric, extract, and dyed fabric against *S. aureus* and *E. coli* bacterial strains reveal significant changes after treatment. The higher effect against activity reveals that this binary colorant has excellent potential to resist bacterial growth (Table 1), which is a positive sign for its potential usage in textile products. Similarly, the antioxidant activities of the extract and dyed fabrics after treatment reveal (Table 1) that the binary colorant has excellent potential and scavenging properties against DPPH radicals, particularly for the dyed fabric after treatment and coloration under statistically selected conditions. Hence, the binary extract from harmal and thuja as a source of natural dye has no harm for global communities, as their combined influence against bacterial and oxidation action is pretty excellent, which is a useful functional characteristic, and hence the extract is appropriate for the global community to use frequently without any fear in textiles and allied fields.

**Figure 5:**
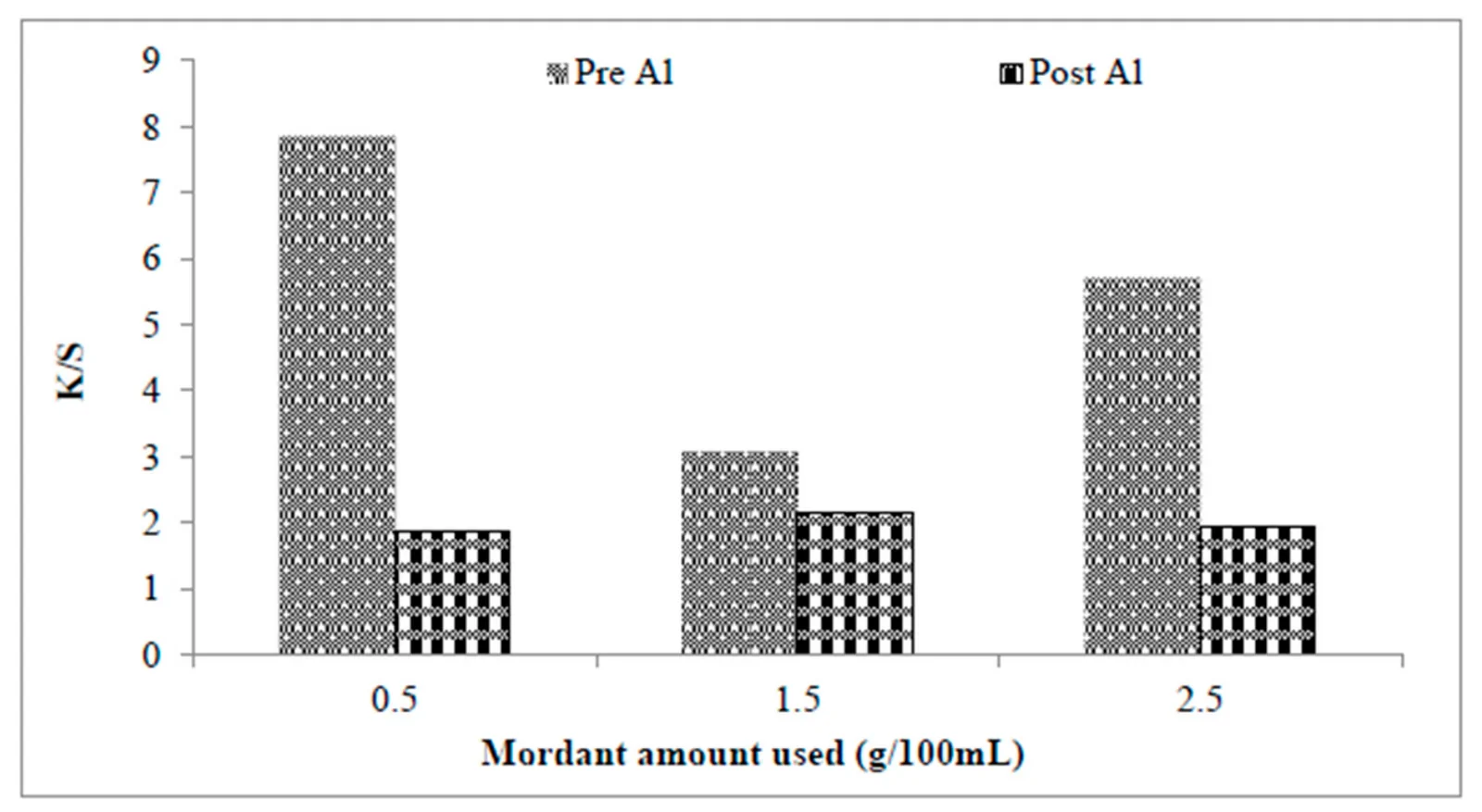
Effect of an Al-acetate solution as a pre- and post-mordant on the dyeing of silk fabric using a binary extract of thuja and harmal.

**Figure 6:**
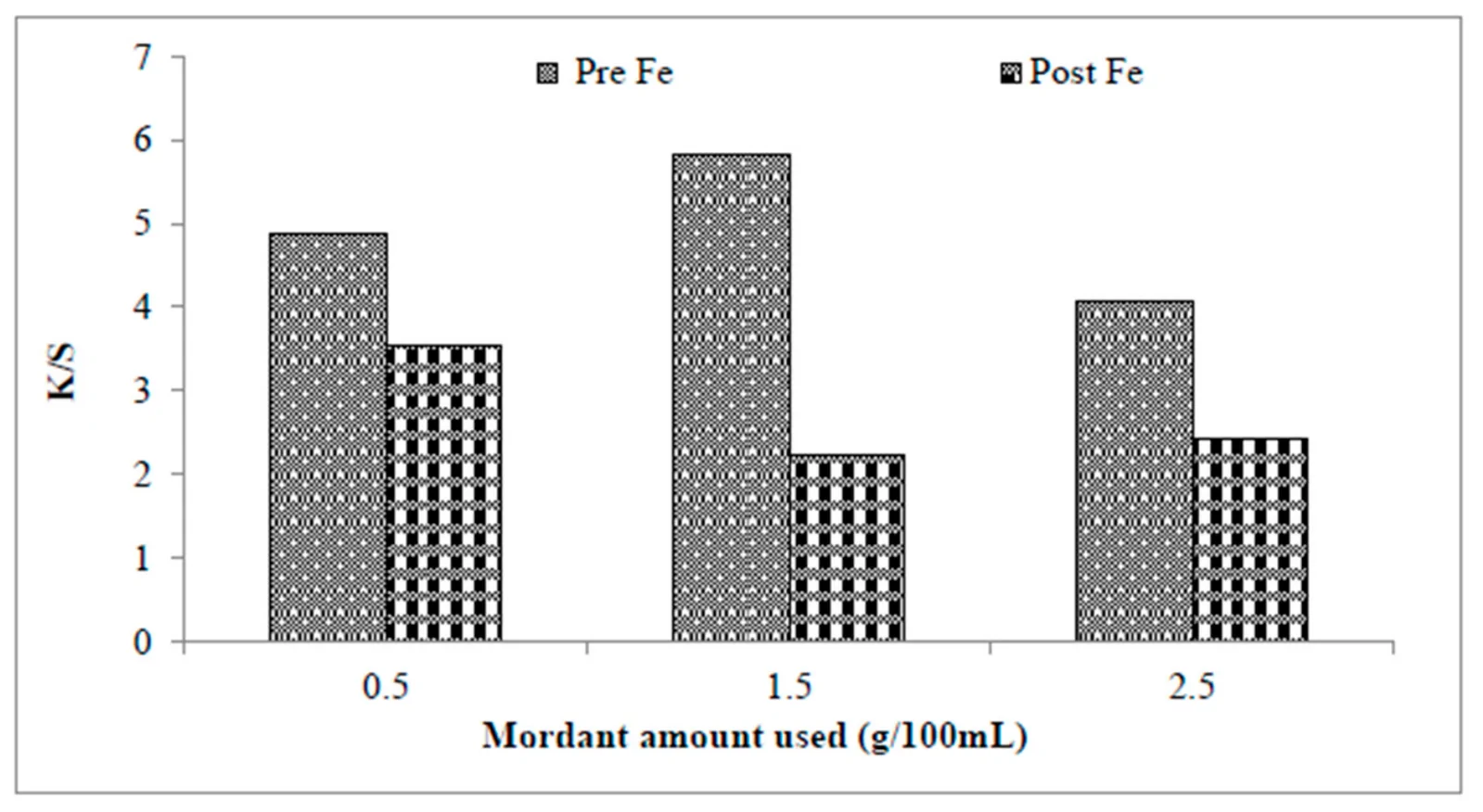
Effect of an Fe-salt solution as a pre- and post-mordant on the dyeing of silk fabric a using binary extract of thuja and harmal.

The addition of metal salts in natural dyeing is vital to overcome fastness issues. These additives are used before and after dyeing of silk with an extract from harmal and thuja to develop colorfast shades. Al^3+^ salt is considered an eco-friendly additive because the Al^3+^ in alum creates a chemical bond with the fabric and binary colorants through firm fixation [25,36]. Its utilization helps to retain the color identity on the fiber with the appearance of a bright hue. Therefore, 0.5% Al^3+^ salt is used for coating the silk fabric followed by dyeing with the binary colorants (Figure 5) and it develops a shade of high strength (K/S = 7.845). After dyeing of silk with a binary colorant from thuja and harmal, the coating of dyed silks with 1.5% Al^3+^ salt provides good shade strength (K/S = 2.16). The shade developed by pre-mordanting is bright yellow in hue with a less reddish tone, having high chromaticity and saturation in color. Similarly, post-mordanted dyed fabric provides a bright yellow shade with less chromaticity and saturation in hue (Table 2). The use of iron mordant is also found to be eco-friendly because Fe^2+^, when it interacts with the fabric and dye, has the potential to change the shade. It can be seen from Figure 6 that before dyeing, the coating of silk with 1.5% Fe^2+^ salt has given the best shade of up to 5.815 (K/S). Similarly, after dyeing of the silk with the binary extract, 0.5% Fe^2+^ salt causes development of a shade of better strength (K/S = 3.54). The pre-mordanted shade is dark yellow with a less reddish tone, with good saturation and hue value (Table 2). The post-mordanted dyed silk on mordanting is much darker yellow in hue with a less reddish tone, having less saturation but a good hue value. This dark shade is due to the utilization of d-orbitals to turn the metal dye complex onto fabric with dye. Normally, a small quantity (1–2%) is sufficient [34]. Hence, before dyeing, use of 0.5% Al^3+^ salt and 1.5% Fe^2+^ salt in dyeing of the silk with the binary extract from thuja and harmal is recommended to get a beautiful shade of high strength. To develop darker shades using statistically selected dyeing conditions usage of lower amounts has not only become economical and sustainable, but has also allowed development of a new colorfast hue.

**Figure 7:**
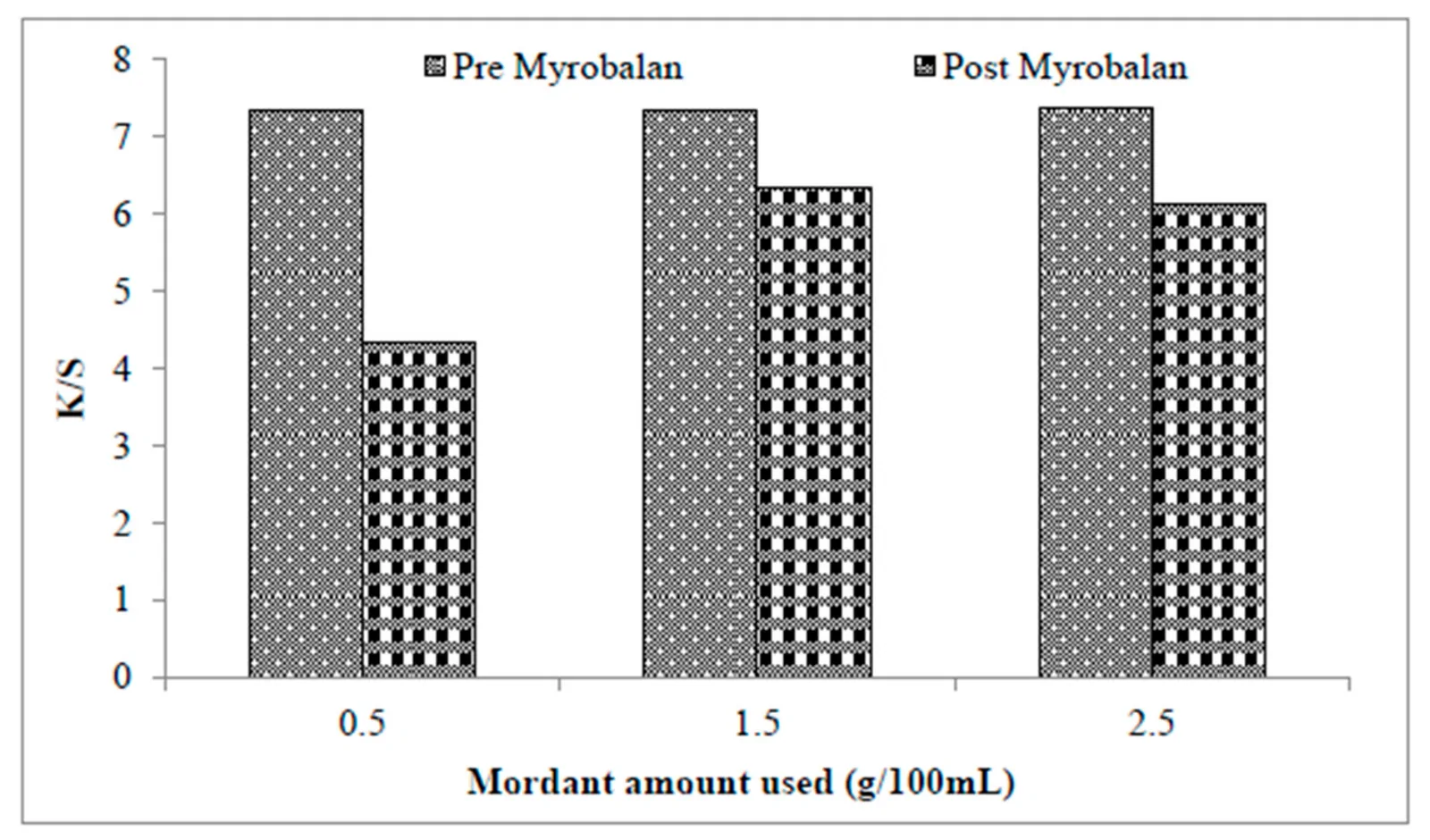
Effect of myrobalan extract as a pre- and post-mordant on the dyeing of silk fabric using a binary extract (thuja and harmal).

Recently, in the last five years, it has been found that plant-based organic extracts with tannins have an eco-friendly nature. These tannins can be used not only to produce soothing shades of a high strength, but also, by overcoating, to help dyed fabrics with enhanced build-up characteristics. Myrobalan is a wonderful bio-mordant enriched with tannins that has excellent functional properties [21,41]. Its usage has made the dyeing process more green, cost-effective, and colorfast. The results in Figure 7 show that before dyeing, 2.5 g/100 mL of myrobalan extract provided the highest yield (K/S = 7.37), whereas after dyeing, 1.5 g/100 mL of myrobalan provided a better yield (K/S = 6.33) on silk. The pre-mordanted shade is a bright yellow shade with a greenish tone with a high chromaticity and saturation value. The post-mordanted shade is a bright yellow shade with a reddish tone with a good saturation and chromaticity value. Thus, the use of bio-mordants before dyeing of silk with a binary extract from thuja and harmal is recommended to get sustainable colorfast shades.

Colorfastness in natural dyeing is the prime demand of the global community for natural dyed products, particularly in textiles. Without any additives, its rating is low, whereas the use of eco-salts or bio-additives has the potential to improve fastness. Using metal salts, a bridge is created via a coordinate covalent bond between the –CONH of fabric (silk) and the dye –OH, which produces stronger, brighter or darker shades [42]. The shade fastness depends upon the nature of the dye, fabric pretreatment (MW), and the mordant type used. The developed metal dye complex with the silk fabric has resisted up to a maximum extent against soaping. Similarly, mordanted dyed silk has also resisted quick heat and light fading [43]. Also, during crocking in both wet and dry conditions, staining has been observed less, which shows the formation of firm, stable, and colorfast shades. The good-to-excellent fastness (Table 3) is due to surface modification of the fabric by MW treatment, dyeing with the binary colorant under statistically selected conditions, and the use of metal or bio-mordants as well as a conjugation system of polyphenolics from a binary colorant coupled with a conjugation system of bio-mordants [17]. Hence, the combined effect of chemicals or bio-mordants in the dyeing of silk with a binary colorant has produced colorfast, stable shades with good properties (Table 3). Hence, the binary colorants produced from thuja and harmal also have good potential to be used as natural alternatives to their synthetic counterparts if used under selected conditions and with eco-friendly mordanting before and after the process. The improved coloration of the silk due to the adopted binary system can be attributed to the complementary adsorption and coordination of chemically diverse phyto-constituents extracted from harmal and thuja. It is important to note that the nitrogen-rich alkaloid species in the harmal extract and the oxygenated phenolic/flavonoid species of the thuja extracts may exhibit different attractions toward silk and mordant species. A possible dyeing mechanism involves various interactions, including hydrogen bonding, electrostatic interactions, and possible coordination with mordant ions, which could collectively influence dye uptake and shade development.

## 4. Conclusions

This research work showed that a binary natural colorant derived from thuja leaves and harmal seeds can be effectively utilized for sustainable silk dyeing through the integration of MW-assisted extraction, statistical optimization, and bio-based surface modification. RSM successfully identified the combined effects of dyeing variables and established optimum conditions of 6 g plant powder, 3 min MW irradiation, pH 3, temperature 75 °C, and 40 min dyeing time, resulting in the highest color strength (K/S = 6.15). Additionally, statistical analysis confirmed that dye bath pH and MW treatment were the dominant factors governing dye uptake, while the predictive model accurately described the experimental response. The findings of the study indicated that the application of bio-mordants and eco-friendly metallic salts significantly enhanced shade development, colorfastness, and functional performance without relying on conventional synthetic dyeing systems. In addition to the improved colorfastness characteristics, the dyed silk showed appreciable antioxidant and antibacterial properties, showing the suitability of the binary plant extract for multifunctional textile applications. So, the findings of our work demonstrate that combining renewable plant resources, MW-assisted processing, and statistical optimization offers an efficient and environmentally responsible strategy for natural silk coloration. The proposed approach reduces processing intensity, improves resource utilization, and supports the development of functional bio-based textiles, providing a practical foundation for future sustainable textile manufacturing and the broader application of natural colorants in advanced textile finishing.

## Figures and Tables

**Table 1:** Antibacterial and antioxidant activity of binary extract (thuja and harmal) and dyed fabrics.

S. No	Extracts		S. No	Fabrics	
	*S. aureus*	*E. coli*		*S. aureus*	*E. coli*
1	24 ± 0.189 mm	25 ± 0.432 mm	3	24 ± 0.371 mm	26 ± 0.545 mm
2	23 ± 0.321 mm	25 ± 0.245 mm	4	21 ± 0.329 mm	27 ± 0.249 mm
Antioxidant					
S. No	SAMPLE	Activity (%)	S. No	SAMPLE	Activity (%)
1	Extract (Irradiated)	83%	4	Dyed Fabric (Non-Irradiated)	90%
2	Extract (Non-Irradiated)	85%	5	Fabric (Irradiated)	32%
3	Dyed Fabric (Irradiated)	87%	6	Control (Non-Irradiated)	31%

1 = Irradiated binary extract, 2 = non-irradiated binary extract, 3 = irradiated dyed fabric, 4 = non-irradiated dyed fabric, 5 = irradiated fabric, 6 = non-irradiated fabric.

**Table 2:** Tonal expressions of dyed silk fabric using a binary colorant from thuja and harmal extract before and after mordanting.

Aluminum Acetate Solution (AAS)						
Mordant concentration	K/S	L*	a*	b*	c*	h
0.5%pre	7.8455	70.32	2.66	29.47	29.59	84.80
1.5% post	2.1649	74.44	6.35	22.35	23.23	74.13
**Iron Sulfate Solution (ISS)**						
1.5% pre	5.8157	63.23	0.35	17.27	17.29	88.84
0.5% post	3.5467	59.41	1.57	13.52	13.61	83.39
**Myrobalan Extract**						
2.5% pre	7.3732	77.54	0.54	29.60	29.61	88.95
1.5% post	6.3349	74.35	2.75	29.34	29.46	84.65

**Table 3:** Evaluation of colorfastness properties of dyed and mordanted silk fabric with binary colorant.

Mordants	Conc (%)	LF	DRF	WRF	WF	Color Shades
Pre-Al^3+^	0.5	5	5	4/5	5	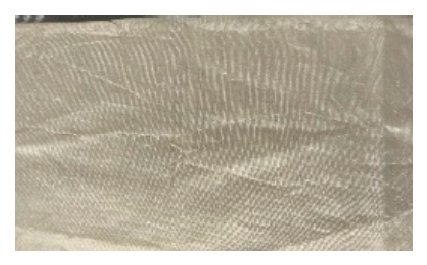
Post-Al^3+^	1.5	5	5	4	4/5	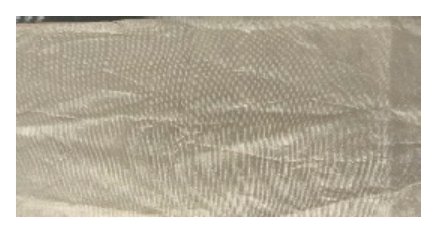
Pre-Fe^2+^	1.5	5	5	4/5	5	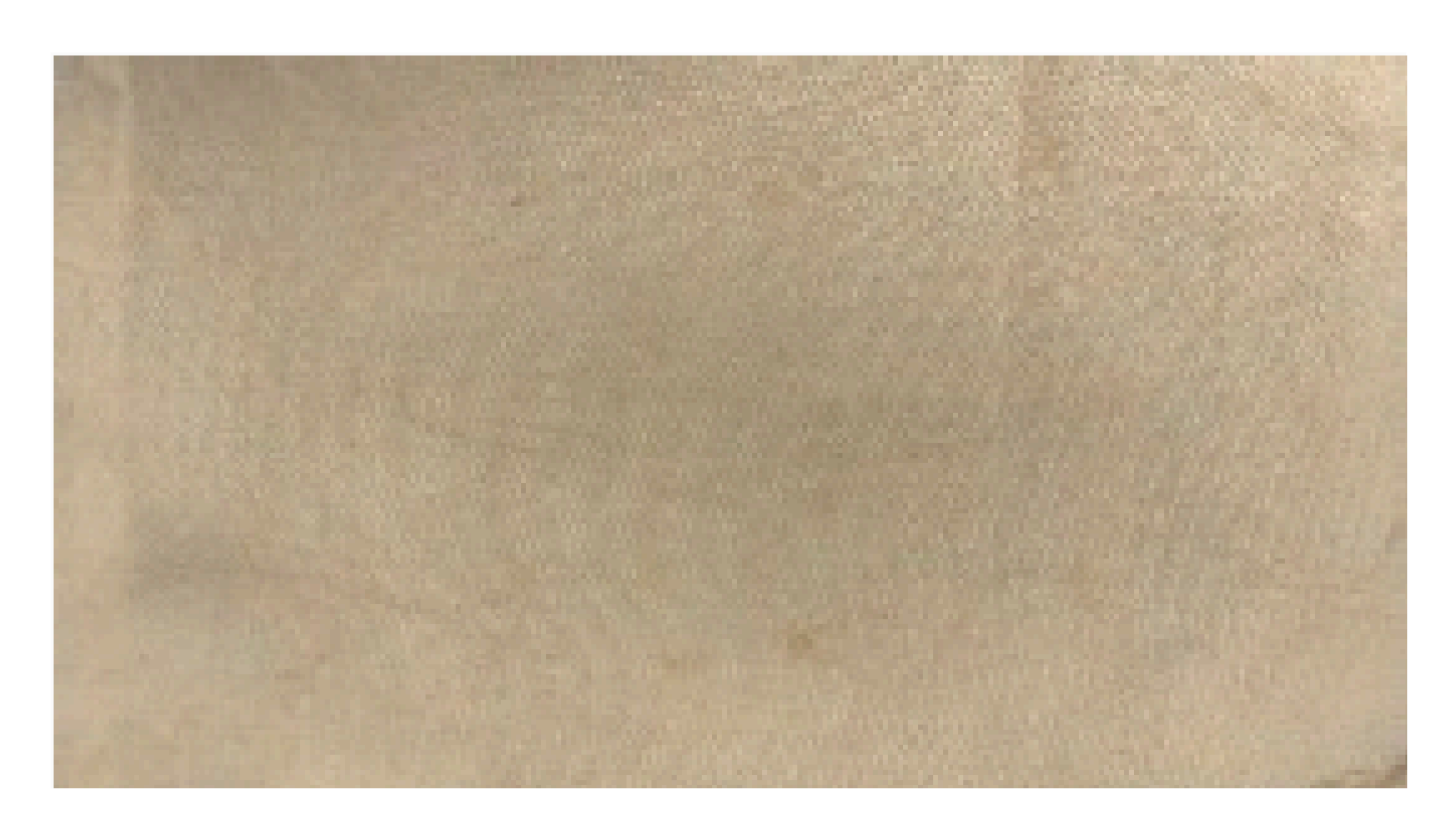
Post-Fe^2+^	0.5	5	5	4	5	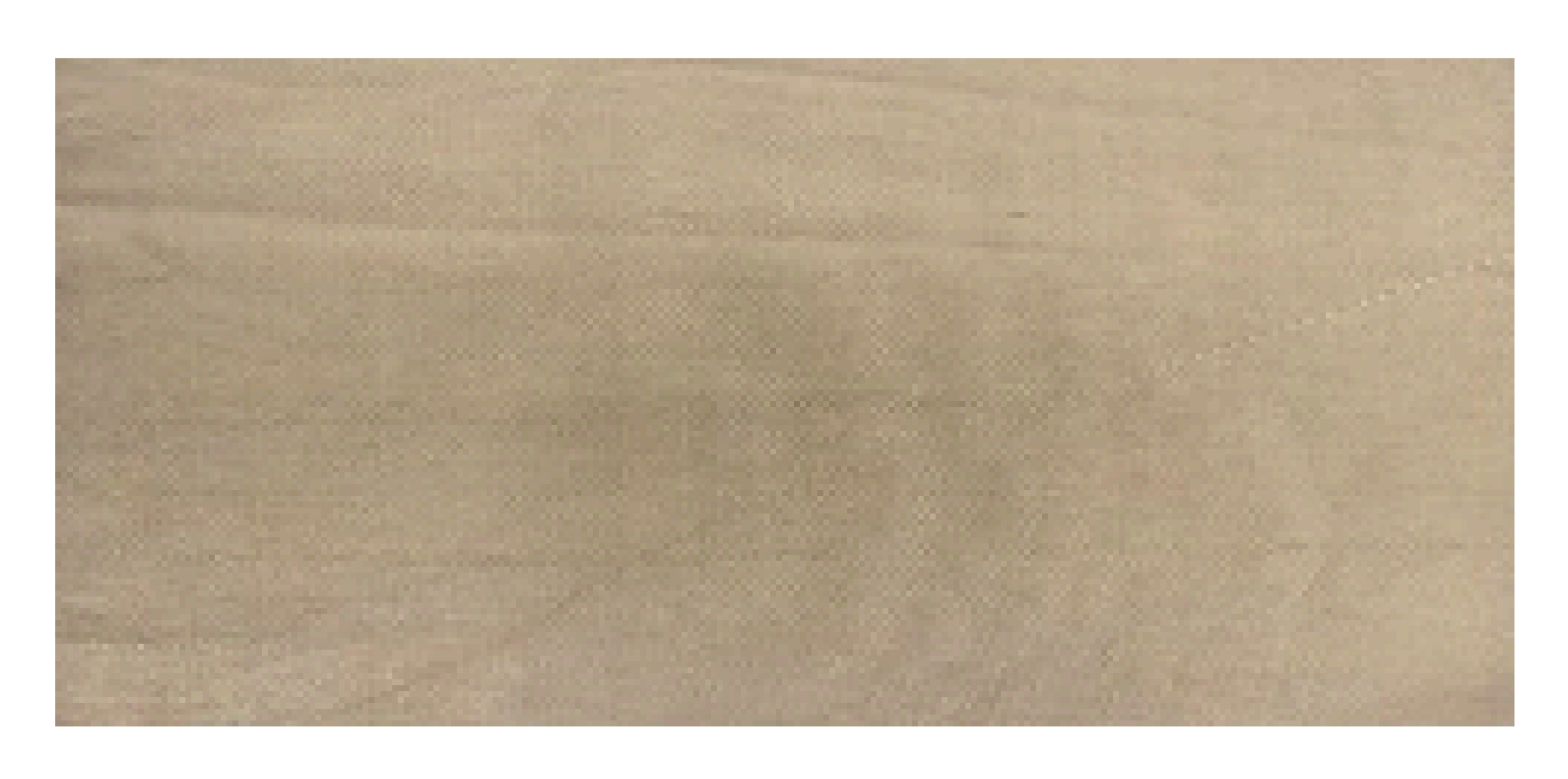
Pre-Myrobalan	2.5	5	5	4/5	5	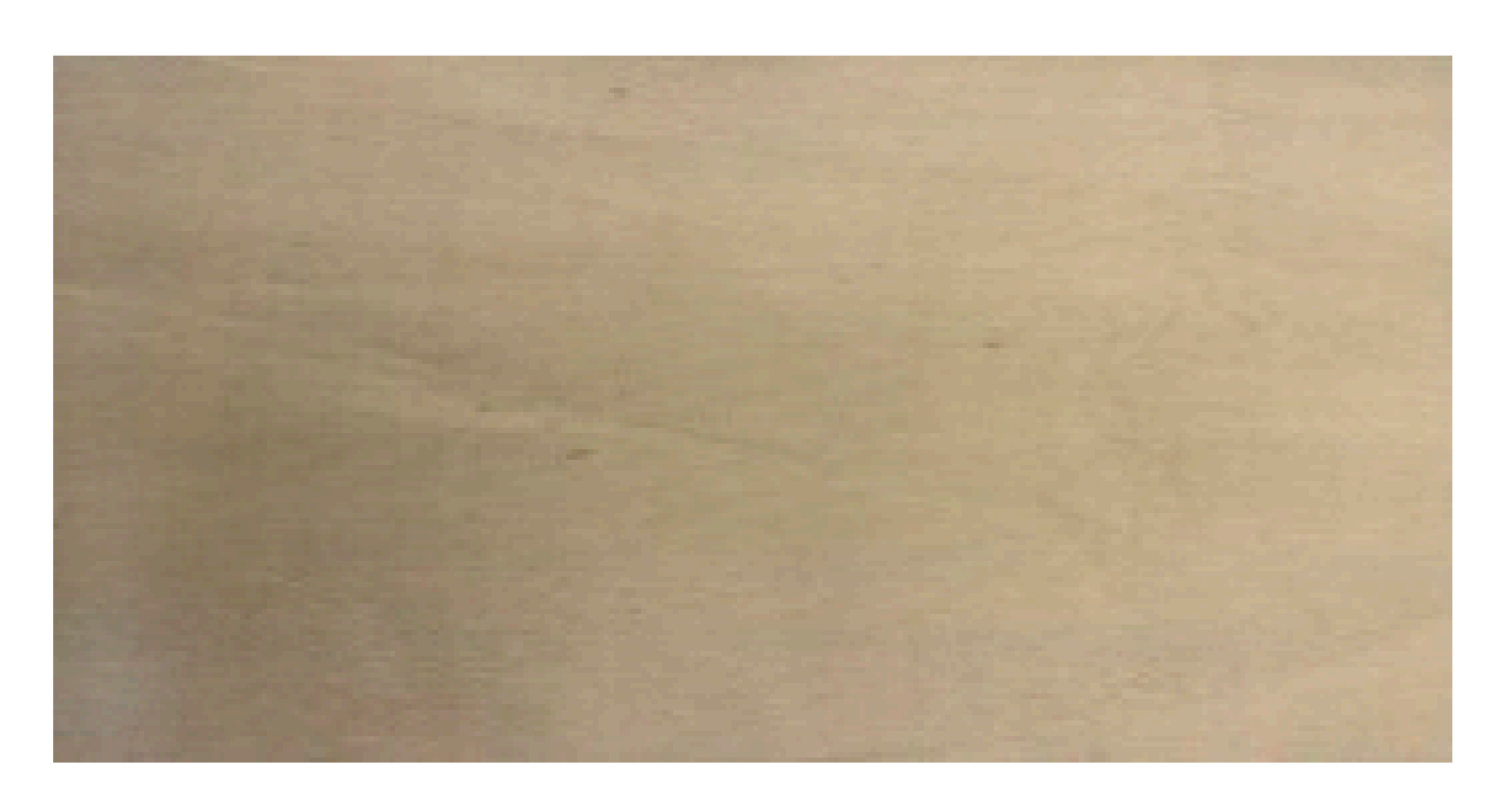
Post-Myrobalan	1.5	5	5	5	4/5	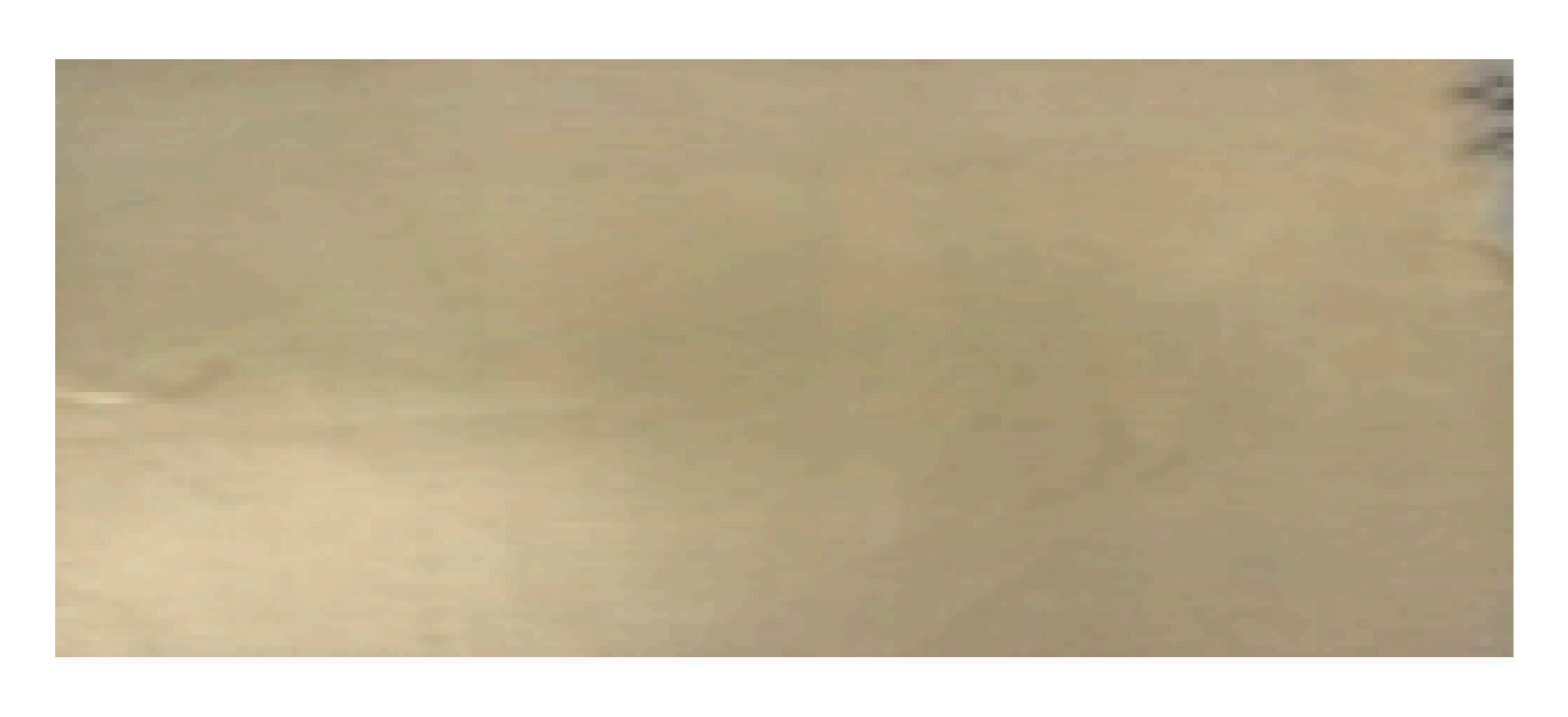

## Data Availability

The work is part of M.Phil studies, so almost every data presented in the paper is available in thesis.

## Supplementary Materials

The following supporting information can be downloaded at: https://www.mdpi.com/article/10.3390/molecules31183253/s1, Figure S1: Technical flowchart for the sustainable dyeing of silk using harmal and thuja binary extract; Table S1: Selection of dyeing variables of silk fabric using binary extract (harmal and thuja); Table S2: Statistical analysis of the dyeing parameters of silk fabric using central composite design.
