# Supplementary material for: Sustainable Dyeing and Mordanting of Silk with a Binary Colorant from Agro-Waste Material
Source: Molecules. 2026 Sep 14;31(18):3253. doi: 10.3390/molecules31183253 (PMC13609825; doi:10.3390/molecules31183253)
Supplement: Supplementary file 1 [file molecules-31-03253-s001.zip › molecules-4492674-supplementary.pdf]

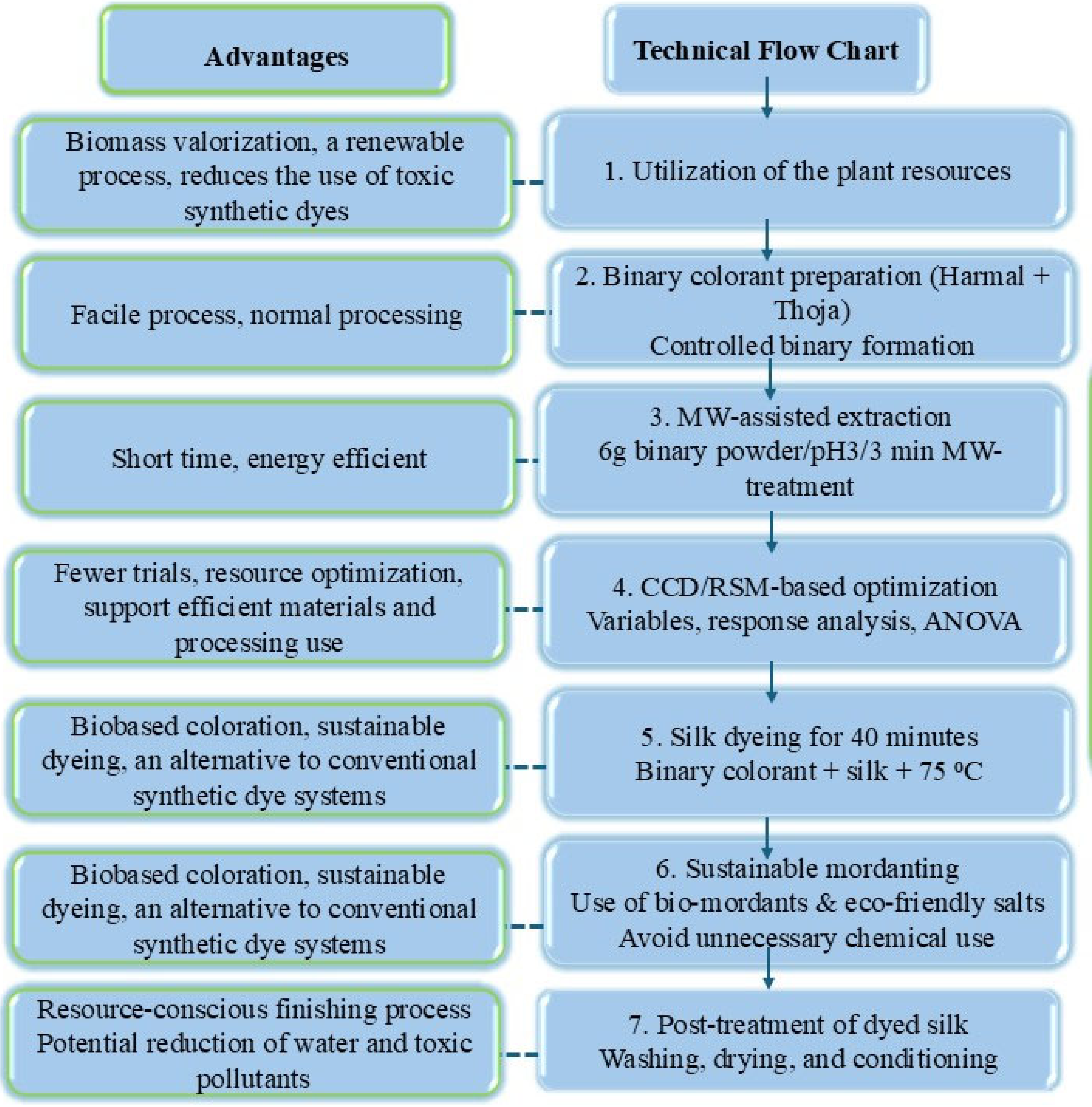

Figure S1. Technical flowchart for the sustainable dyeing of silk using harmal and thuja binary extract

Table S1. Selection of dyeing variables of silk fabric using binary extract (harmal and thuja)

Radiation time (min.)	Powder (g)	Temperature (°C)	Time (min.)	pH	K/S
2	4	65	30	6	4.70
4	4	65	30	4	3.02
2	8	65	30	4	5.01
4	8	65	30	6	3.81
2	4	85	30	4	3.96
4	4	85	30	6	4.76
2	8	85	30	6	3.54
4	8	85	30	4	5.49
2	4	65	50	4	3.77
4	4	65	50	6	2.81
2	8	65	50	6	3.15
4	8	65	50	4	4.21
2	4	85	50	6	2.70
4	4	85	50	4	4.52
2	8	85	50	4	5.74
4	8	85	50	6	4.63
1	6	75	40	5	3.49
5	6	75	40	5	4.68
3	2	75	40	5	4.50
3	10	75	40	5	4.48
3	6	55	40	5	4.54
3	6	95	40	5	3.64
3	6	75	20	5	4.33
3	6	75	60	5	5.10
3	6	75	40	3	6.15
3	6	75	40	7	1.86
3	6	75	40	5	5.20
3	6	75	40	5	5.01
3	6	75	40	5	4.89
3	6	75	40	5	6.04
3	6	75	40	5	4.74
3	6	75	40	5	4.35

Table S2. Statistical analysis of the dyeing parameters of silk fabric using central composite design

Source	DF	Adj SS	Adj MS	F Value	P Value
Model	20	26.1043	1.3052	4.37	0.010
Linear	5	14.4562	2.8912	9.68	0.01
Radiation time	1	2.6263	2.6263	8.79	0.014
Powder	1	0.0226	0.0226	0.08	0.789
Temperature	1	0.2910	0.2910	0.97	0.347
Time	1	0.7511	0.7511	2.52	0.144
pH	1	12.8472	12.8472	43.02	0.000
Square	5	7.1975	1.4395	4.82	0.017
Radiation time* Radiation time	1	2.6119	2.6119	8.75	0.014
Powder*powder	1	1.1690	1.1690	3.91	0.076
Temperature*Temperature	1	2.6153	2.6153	8.76	0.014
Time*time	1	0.6075	0.6075	2.03	0.184
pH*pH	1	2.9687	2.9687	9.94	0.010
2-way interaction	10	11.6891	1.1689	3.91	0.021
Radiation time*powder	1	1.9342	1.9342	6.48	0.029
Radiation time *Temperature	1	6.3197	6.3197	21.16	0.001
Radiation time *Time	1	1.1119	1.1119	3.72	0.082
Radiation time *pH	1	3.5695	3.5695	11.95	0.006
Powder*Temperature	1	0.8619	0.8619	2.89	0.120
Powder*time	1	3.0978	3.0978	10.37	0.009
Powder*pH	1	5.1384	5.1384	17.21	0.002
Temperature*time	1	3.0477	3.0477	10.21	0.010
Temperature*pH	1	3.1037	0.1037	10.39	0.0090
Time *pH	1	0.1423	0.1423	0.48	0.506
Error	10	2.9863	0.2986	___	____
Lack-of-fit	5	1.3780	0.2756	0.86	0.565
Pure Error	5	1.6082	0.3216	___	____
Total	30	29.0906	___	___	___
**Model summary**	**S**	**R-Sq**	**R Sq (adj)**	**R Sq (pred)**	___
___	0.546469	89.73%	---------------	--------	___
